# Fatal Left Ventricular Aneurysm in a 13 Years Old Male Child: A Case Report

**DOI:** 10.4314/ejhs.v31i4.26

**Published:** 2021-07

**Authors:** Tsion Tilahun, Elias Kedir, Beza Eshetu

**Affiliations:** 1 Department of Pediatrics and Child Health, Jimma University; 2 Department of Radiology, Jimma University

**Keywords:** left ventricle, aneurism, diverticulum, cardiogenic shock, case report

## Abstract

**Background:**

A pouch protruding from the free wall of the left ventricle may be either a congenital ventricular diverticulum or congenital ventricular aneurysm. Congenital ventricular aneurism is a ventricular protuberance which is a kinetic or dyskinetic and on histology is predominantly fibrous tissue with no organized myocardium. Common clinical presentations of congenital ventricular aneurism are arrhythmia, rupture and heart failure.

**Case Detail:**

A 13 year old patient presented with shortness of breath, fever, orthopnea of two pillows and paroxysmal nocturnal dyspnea of one week duration. Echocardiography revealed cystic mass seen at the apex of the heart communicating with left ventricle, with communicating defect and flow on color Doppler study. CT scan showed ventricular aneurism at the apex. The patient was managed for heart failure and passed away after few hours' of establishing diagnosis.

**Conclusion:**

Congenital ventricular aneurysm is a rare condition which needs careful diagnosis for subsequent management.

## Introduction

A pouch protruding from the free wall of the left ventricle may be either a congenital ventricular diverticulum (CVD) or congenital ventricular aneurysm (CVA). CVD and CVA are distinct entities with different histology, presentation, associated defect and prognosis (1). CVD is used to describe an out- pouching from a ventricle which contracts synchronously with that chamber and histologically contains all three layers of the ventricular wall (endocardium, myocardium, and pericardium). CVA is a ventricular protuberance which is akinetic or dyskinetic and on histology is predominantly fibrous tissue with no organized myocardium (2). We report a case of CVA in a 13 years old adolescent who presented to our hospital.

## Case Details

This was 13 years old adolescent who was previously healthy presented with shortness of breath, fever, orthopnea of two pillows, paroxysmal nocturnal dyspnea and productive cough of one week duration. He has no history of trauma, any previous similar complaint or admission to health facility.

Physical examination revealed a well-nourished child in respiratory distress. His pulse rate was 120 beats/minute, regular and full in volume; respiratory rate was 36 breaths/min, temperature was 36.5°C and blood pressure was 100/64mmHg. On chest examination, he had decreased air entry over left lower third of the chest and bronchial breath sound on the right lower third of posterior chest. Precordial examination showed bulged precordium; apical heave and systolic thrill at the apex; point of maximal impulse was at the 6th intercostal space at mid clavicular line and had grade IV holosystolic murmur at the apex. Liver was palpable 3cm below right costal margin and tender. Other findings were unremarkable.

With an impression of heart failure secondary to chronic rheumatic valvular heart disease and pneumonia, he was investigated with chest x-ray which showed cardiomegaly. CBC showed thrombocystosis (502,000/mm3), ESR was 100 mm in the 1^st^ hour and ASO was non-reactive.

**Figure 1:**
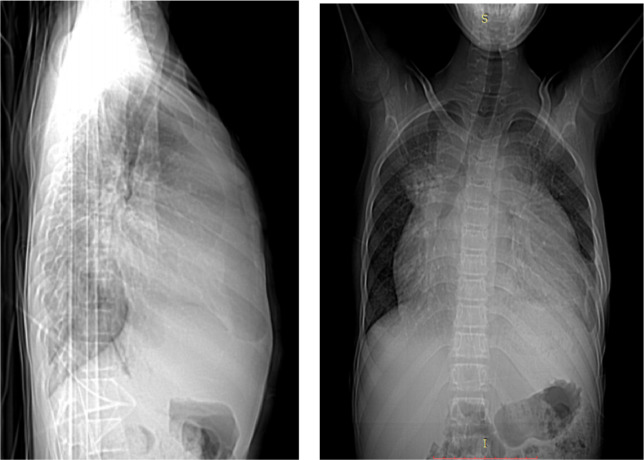
Chest CT-scan showing huge cardiomegaly

**Figure 2:**
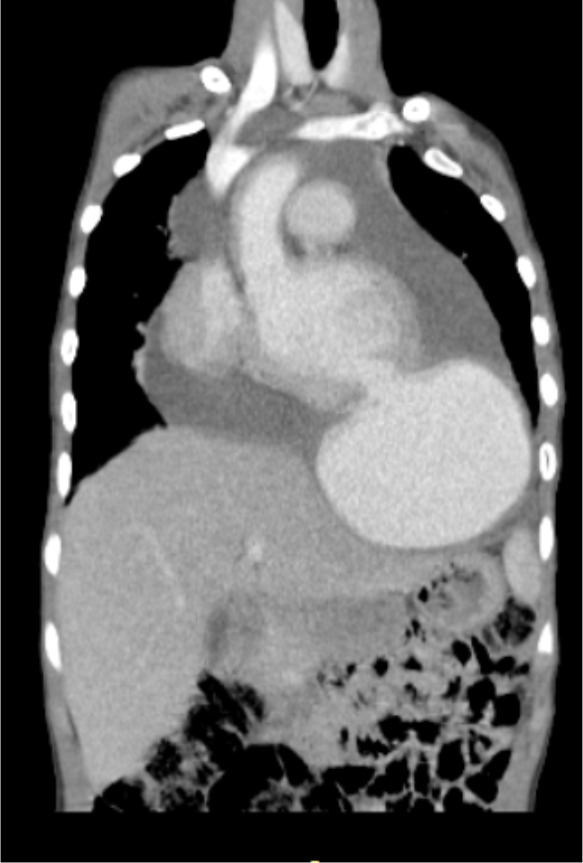
CT-scan of the heart with a communication of the left ventricle with aneurysm

With the above diagnosis, he was put on intravenous ceftriaxone and furosemide. Echocardiography was done after 3 days of admission and revealed cystic mass seen at the apex of the heart communicating with left ventricle, with communicating defect. The cyst has fluid and flow on color Doppler study. CT scan was also done and showed ventricular aneurism at the apex which measures 8.51 cm x 8.73 cm × 9.78 cm (Figures 1, 2).

The patient showed no much improvement and on 3^rd^ day of admission, he suddenly collapsed and started to have fast breathing, diaphoresis and change in mentation. His blood pressure became unrecordable and pulse was feeble; he had cold extremity and delayed capillary refill. With an additional assessment of cardiogenic shock secondary to ruptured aneurism, normal saline 10 ml/kg was given and he was put on adrenaline drip but he deteriorated and arrested for which cardiopulmonary resuscitation (CPR) was done but unfortunately he passed away with the likely cause of death of cardio respiratory failure.

## Discussion

Left ventricular aneurysm is defined as a distinct area of abnormal left ventricular diastolic contour with systolic dyskinesia or paradoxical bulging as visualized by ventriculography (3). Congenital aneurisms have saccular expansion of the ventricular wall, with a wide collar communicating with the ventricular cavity. However, diverticula are elongated and have a narrowed neck. Most frequently, location of LVA is the LV apex (28%) and the perivalvular area close to the mitral valve (49%) (4).

There are two groups of left ventricular aneurysms in children, acquired aneurysms (traumatic) and congenital aneurysms (5). Congenital aneurysms of the left ventricle are uncommon entities. The largest series of these kinds of aneurysms that we found in the literature is 6 cases over a period of 11 years; in 2006(5). LVA usually results from myocardial infarction. Other etiologies of LVA include hypertrophic cardiomyopathy, Chagas' disease, sarcoidosis and idiopathic dilated cardiomyopathy. Other case reports have showed chemotherapy induced left ventricular aneurism (3). In our patient, there was no any identifiable risk factor.

Congenital LVA or diverticula are often asymptomatic and usually found coincidentally during diagnostic procedures performed for other reasons. This may explain the late diagnosis of LVA, on average, at the age of 31.5 years. The most frequent clinical presentation in LVA includes arrhythmias (18.4%), embolic events (5.4%), rupture (4 %), congestive heart failure (21.5%) and angina pectoris rarely. The rupture in general appears to be a problem of the younger age groups as the median age at the time of rupture was in the perinatal period (4). Patients may present with valvular pathology as those aneurysm in a perivalvular position may be associated with valvular insufficiency, prolapse or perforation (1). In our case, the patient presented with heart failure. Congenital LVA might be associated with other cardiovascular malformations such as ventricular diverticula, septal defects, malformation of the thoraco-abdominal aorta and mitral insufficiency or could be isolated (5). In our case, we didn't detect any other associated malformation.

Contrast ventriculography is considered as gold standard for the diagnosis of LVA; 2D echocardiogram also has high specificity and sensitivity (3). With regard to treatment, all authors agree with the surgical treatment when the aneurysm is symptomatic. However, controversy exists regarding the asymptomatic aneurysms; some authors recommend a systematic resection to prevent complications such as thrombosis, rupture of the aneurysm, and left ventricular failure while others do not recommend routine resection due to the elevated mortality (5). Treatment has to be individually tailored and depends on clinical presentation, accompanying abnormalities and possible complications. Options include surgical resection (especially in symptomatic patients), anticoagulation after systemic embolization, radiofrequency ablation or implantation of an implantable cardioverter defibrillator in the case of symptomatic ventricular tachycardias, and occasionally combined with class I- or IIIantiarrhythmic drugs. Cardiac death usually occurs in childhood, is significantly more frequent in LVA patients and caused by congestive heart failure in most of the cases (4). Unstable pathologies have a high risk of rupture and survival from this is rare (5). In our case the patient should have been investigated well with ECG and autopsy should have been done for confirmation of diagnosis.

Congenital ventricular aneurysm is a rare condition which needs careful diagnosis for subsequent management. Early diagnosis and strict follow up in the hospital with ECG in ICU could have been done for the patient. Pediatric cardiologist should have been consulted which was not available in our setting.

**Ethical consideration**: Verbal consent was obtained from the parents before writing this case report.
